# Outpatient group therapy for post-COVID patients - a naturalistic feasibility study of a face-to-face and online group concept

**DOI:** 10.3389/fpsyt.2024.1500210

**Published:** 2024-12-17

**Authors:** Verena Zimmermann-Schlegel, Nadine Gronewold, Sandra Stengel, Mechthild Hartmann, Uta Merle, Hans-Christoph Friederich, Beate Ditzen, Jonas Tesarz

**Affiliations:** ^1^ Department of General Internal Medicine and Psychosomatics, Heidelberg University, Heidelberg, Germany; ^2^ Institute of Medical Psychology, Centre for Psychosocial Medicine, Heidelberg University, Heidelberg, Germany; ^3^ Institut für Arbeitsmedizin (ifa), Institute for Occupational Medicine, Baden, Switzerland; ^4^ Department of General Practice and Health Services Research, Heidelberg University, Heidelberg, Germany; ^5^ Department of Internal Medicine IV, Heidelberg University, Heidelberg, Germany; ^6^ DZPG (Deutsches Zentrum für Psychische Gesundheit) - German Centre for Mental Health - Heidelberg, Germany; ^7^ Department of Psychosomatic Medicine and Psychotherapy, University Medical Center of the Johannes Gutenberg University Mainz, Mainz, Germany

**Keywords:** online therapies, post-COVID, group therapies, feasibility, fatigue, coping

## Abstract

**Background:**

A significant number of individuals diagnosed with SARS-CoV-2 continue to suffer from persistent symptoms, a condition commonly referred to as Post-COVID syndrome (PCS). The most common manifestations are fatigue, post-exertional malaise, respiratory problems and cognitive deficits due to the lack of a causal treatment, therapeutic options remain symptom oriented. The aim of this study was to develop a low-threshold group therapy concept for patients with PCS and to test its feasibility in face-to-face and online format.

**Method:**

An interprofessionally oriented group therapy concept for patients with PCS was developed and a treatment manual was established. The concept comprises eight weekly sessions of 90 minutes each, during which the management of fatigue, stress intolerance and other symptoms are addressed and coping strategies are discussed and developed. The group therapy was conducted alternating in face-to-face and online format and evaluated via questionnaires.

**Results:**

A total of 57 patients, most of them with severe limitations due to PCS, took part in the groups (n=36 online; n=21 face-to-face). The group offer was requested and accepted in both the face-to-face and online formats, and was predominantly evaluated as beneficial. Of particularly value was the opportunity to engage with peers who share similar experiences.

**Conclusion:**

The interprofessional, integrative psychotherapeutic/psychoeducational group therapy is safe, accepted and is predominantly rated as helpful by participants. It should be carried out in online formats for patients with PCS who are limited in mobility. Controlled studies are necessary to further evaluate the proposed concept and its integration into the care landscape.

## Introduction

It is estimated that up to 10% of individuals infected with SARS-CoV-2 experience persistent symptoms for months after initial infection, a condition known as post-COVID syndrome (PCS) (1). According to the WHO definition, PCS is present if symptoms persist for a minimum of three months following probable or confirmed SARS-CoV-2 infection, last for a minimum of two months, and cannot be explained by an alternative diagnosis (2). The English NICE (National Institute for Health and Care Excellence) guidelines distinguish between “persistent COVID-19 symptoms” associated with SARS-CoV-2 infection (present for a period of 4-12 weeks following infection) and post-Covid syndrome (>12 weeks), with both stages collectively referred to as “Long-Covid” (3).

The primary symptoms of PCS in the majority of patients are fatigue and exercise intolerance, with the specific presentation of so-called “post-exertional malaise” (PEM). PEM is defined as a transient or permanent worsening of symptoms following physical, mental or even emotional stress. Additionally, numerous patients also report cognitive impairments, including difficulties of concentration and memory, and a phenomenon known as “brain fog”. Other common symptoms include respiratory difficulties and headaches (4).

Psychological symptoms include depression, anxiety, post-traumatic stress symptoms and sleep disorders. Recent studies show that mental stress, and in particular depression, also increases the risk of cognitive deficits which makes their examination and treatment urgently necessary (5). Overall, patients suffering from PCS report a reduced quality of life (6–8). The impact of the symptoms on an individual´s ability to function can range from minor impairments in everyday life to complete immobility and the necessity for care. Some patients also find themselves in precarious financial situations as a result, which in turn acts as an additional psychosocial stress factor that exacerbates symptoms (4).

A number of hypotheses have been proposed regarding potential biological etiologies, including persistent tissue damage, endothelial dysfunction, viral components, chronic inflammation and autoimmune reactions (9). There are also heterogeneous models for the genesis of psychological symptoms, such as the disturbed regulation of neurotransmitters due to pandemic and infection-related stress factors, microglial activation and others (4, 10, 11).

Despite the uncertainty surrounding the somatic causes of PCS, a holistic, bio-psycho-social understanding of PCS posits a bidirectional relationship between psychological and somatic stress. In addition to the psychological consequences (depression, anxiety, sleep disorders) of PCS, which are understood as a consequence of the impairment caused by the persistent and sometimes disabling symptoms, there is compelling evidence that pre-existing mental illnesses or stress factors prior to SARS-CoV-2 infection also significantly increase the risk of developing PCS (12).

There is currently no causal therapy available for PCS, although a large number of interventional/medical approaches are being tested (13). From a holistic perspective, symptom-oriented, supportive forms of treatment are clearly indicated, and appear to be efficacious (4, 14).

Current approaches to this type of supportive therapy aim to alleviate symptoms and prevent chronicity by changing dysfunctional coping strategies (15). The focus is to enhance personal resources, facilitate appropriate coping mechanisms and to manage limited energy reserves. The aim is to avoid the experience of excessive demands or inadequate avoidance of activity, which could potentially lead to symptom chronicity (15, 16).

Multimodal therapies are based on the established approaches for the treatment of chronic fatigue syndrome, which has been treated in a supportive, symptom-oriented manner with cognitive behavioral therapy and adapted training for many years now (15, 17). Psychotherapeutic (co-)treatment is indicated in particular if there is a psychological comorbidity, restrictions exist that reduce the possibilities of coping with everyday life or the subjective stress is so high that the quality of life is additionally restricted (4, 8, 18).

Group therapy formats offer the opportunity to share one’s experience with others, to learn about others’ coping skills, and to develop alternative coping strategies together. This helps patients to feel less isolated and helpless (19). By promoting resources, they can experience themselves as more self-effective actors, which can reduce psychosocial stress. This is also shown by studies on psychotherapeutic and supportive interventions for other (chronic, somatic) illnesses with positive results *and has now also been shown to be effective for patients with PCS* (20–22).

The focus of the group intervention presented here is initially on psychoeducational elements and sharing experiences. The interaction between psychological stress and symptoms, techniques for stress regulation and the topic of resources and resource activation need to be addressed. The aim is to reduce uncertainty, strengthen self-efficacy and support coping with PCS. An important component of the therapy is destigmatization of psychosomatic aspects of PCS and to establish and communicate a biopsychosocial model of the disease.

The therapists/group leaders create a safe and supportive environment and help patients to deal with illness related stress and psychosocial burden and find a constructive way of dealing with it (23). Group leaders encourage sharing and interaction within the group in order to use collective knowledge and develop coping strategies together, thus actively participating in their recovery.

Furthermore, it is desirable to make group participation accessible to those patients who cannot reach a practice or clinic for logistical reasons or who cannot leave their homes due to the severity of their illness. Several studies of digital support therapies for PCS are underway or planned (24). Digital services have been shown to be feasible and effective and offer a flexible and accessible option that ensures more patients can benefit from therapeutic interventions regardless of their location or limitations (25, 26).

Although there are many different therapeutic approaches, patients with PCS remain underserved due to the aforementioned difficulties. It is therefore imperative to develop and test concepts that are both feasible and accepted in broad-based care. The study presented in this manuscript is a feasibility study on a group treatment concept for patients suffering from PCS. The hypothesis to be evaluated is that group therapy with a predominantly psychoeducational and supportive approach is feasible and potentially helpful for patients with PCS.

## Method

### Recruitment and preparation

Patients who attended the outpatient Clinic for PCS at Heidelberg University Hospital were informed about the possibility of participating in the group therapy through personal information and flyers. Information about the group was also shared via a regional PCS network. The high level of demand from patients inevitably led to the development of significant waiting times. The inclusion criterion for group participation was the presence of PCS according to WHO criteria (see above). The group therapies were conducted in succession, with recruitment completed for one format before the other was offered. Patients were provided with detailed information about both face-to-face and online therapy formats and were allowed to choose their preferred option without any pre-selection by the research team. This approach ensured that participants could select the format most aligned with their personal circumstances and preferences.

A preliminary assessment (by telephone) was held with all interested patients, in which they were asked about their symptoms and medical history, and were also informed about the setting, the content of the group sessions and the accompanying questionnaire evaluation. In the online format, the preliminary interviews were supplemented by a short “tech-check” in which the technical procedure was explained and the handling of possible technical problems was discussed. In addition, the necessity of handling the personal information of fellow patients confidentially was explained, which in the case of the online group includes participation in a protected space (without the presence of third parties).

Patients were considered unsuitable for the group if they were suffering from symptoms that were not associated with PCS or if they suffered from conditions which required immediate treatment such as delusional disorders or major depressive disorders with suicidal ideation. These patients were then given a recommendation for more specific therapy.

### Intervention

The target group size was set at 8 to a maximum of 12 patients. On the one hand, the group size should be small enough to allow for sufficient intimacy, individual attention and personal interaction between the group members. On the other hand, it should be large enough to offer sufficient diversity of perspectives and a broad range of experiences. The group therapy comprised a total of 8 sessions plus a refresher session.

The therapeutic approach was informed by prior consultations with approximately 430 PCS patients treated as outpatients in our psychosomatic and specialized departments. These consultations highlighted recurring challenges, including managing fatigue and stress intolerance, as well as the psychological impact of these symptoms on professional and familial roles. Patients also emphasized the importance of peer support and the need for reliable information on treatment options. In addition, a literature review was conducted on psychosocial PCS interventions. The procedure for the meetings was worked out as a manual, each session was assigned an overarching theme (table attached as **Supplementary Table S1**). A refresher session was offered two months after the end of each eight-week intervention.

The groups were led by a psychological psychotherapist and a physician from the field of internal medicine. The input of both a medical doctor and a psychologist was of particular importance in this context. The group leaders were able to represent different aspects of this complex illness and medical questions could also be clarified. The addressing of uncertainties associated with medical questions constituted a necessary precondition for engaging with topics such as coping and personal resources.

The sequence of events in each session was predetermined and unchanging. The introduction was structured around a concept known as the “flashlight” or “state of mind round”. Subsequently, educational components were integrated with interactive phases. The content was structured such that it alternates between brief, themed keynote speeches delivered by the therapists and an open exchange between participants, which constitutes the majority of the group sessions. In order to facilitate the psychoeducational content, simple illustrations were employed in order to visualize the key information. Each session concluded with a breathing or imagination exercise.

Given that patients typically arrived at treatment with a predominantly somatic understanding of their illness, a session was held at an early stage of the cycle in collaboration with a medical doctor from the field of PCS. This provided participants with access to the latest knowledge on the topic and the opportunity to pose their own questions. A session with a physiotherapist was held at the end of the group cycle. This introduced breathing therapy techniques and exercises, which are designed to enhance physical performance in a gradual and controlled manner.

In addition to the group content described above, patients were offered the opportunity to contact the group leader if they have had any specific social law issues, such as questions about sick leave or reintegration into their workplace. In such cases, an individual social work consultation was arranged.

### Questionnaires//assessment

Clinical and sociodemographic data were collected at baseline. The ´PCS score´ was used to assess disease burden (27). ´PCS score´ is a clinical tool for quantifying the severity of symptoms in patients suffering from post-COVID syndrome. This questionnaire surveys the various common symptoms and takes a weighted factor into account when calculating the total. Additional clinical data (e.g. PHQ-9,GAD-7, SF-12) were collected but not presented in this manuscript in order to keep the focus on feasibility and implementation. After the end of the group, the participants’ subjective assessment of the course of their symptoms was surveyed using the PGI-C (Patients Global Impression of Change) as a standardized instrument (7-point Likert scale: very much improved, much improved, minimally improved, no change, minimally worse, much worse, very much worse) (28).

The evaluation form asked about the different components of the group sessions, such as symptom management, perceived benefits of psychoeducational content and peer support with regard to the perceived benefits (How much did you benefit from the following contents of the group therapy)?. Patients could rate on a numeric rating scale (0-10; 0=not helpful at all…. 10=very helpful).

Open questions were asked at the end of the survey for feedback.

### Statistical analysis

Data were analyzed in SPSS using descriptive statistics (frequency, percentage, mean and standard deviation) and inferential statistics (T-test, Chi-square Test) to assess differences between groups. Due to skewed distribution, the results of the evaluation were presented using the median (interquartile range). The Mann-Whitney U test was used to compare groups.

Qualitative analysis was performed deductively on the basis of Yalom’s effectiveness factors for group therapy and additionally inductively on the basis of the available data material.

## Results

### Description of the participants

A preliminary telephone assessment was conducted with 68 interested patients. Out of them 57 patients could be enrolled, of which 36 took part in 3 online group rounds and 21 took part in 2 group rounds face-to-face. Reasons for non-participation (n=11) were inappropriate time of the sessions (n=3), patients felt too ill to participate (n=5), were in rehab at the time of the group (n=1) or were no longer interested (n=2).

A total of 43 participants took part in the post assessments, corresponding to a response rate for the study of 75% (n=43/57). Main reason for non-participation was inability to complete the questionnaires due to concentration difficulties.

Baseline characteristics are shown in **Table 1**. There were no differences between the participants in the online group and the face-to-face group in terms of age, gender and severity of the disease as measured by the ´PCS Score`. However, the participants in the online group reported a longer duration of illness (14.4 vs 7.7 month, p=0.001).

**Table 1:** Baseline characteristics.

	total (n=57)	Online (n=36)	Face-to-face (n=21)	p
Age (yrs)	45.7 ± 13.5 yrs	44.5 + 12.3 yrs	47.8 + 15.5 yrs	.207
Sex (% female)	86%	94%	83%	.110
Duration of symptoms (month)	12.0 ± 7,1	14.4 + 7,0	7.7 + 5.3	.001*
On Sick leave	88%	91%	80%	.234
PCS-Score^a^	29.9 ± 10.9 (n=47, 10 missing)	31.2 + 11.1 (n=29)	27.9 + 10.4 (n=18)	.149

^a^PCS-Score: ≤10.75 No/mild PCS; >10.75 or ≤ 26.25 moderate PCS; >26.25 severe PCS *p-values considered statistically significant.

All participants were clearly limited in their ability to cope with everyday life. Most patients (n=50, 88%) were still on sick leave at the time the group was conducted. No patients needed help with personal hygiene but 28% of participants stated that they needed daily assistance from family members to carry out daily tasks (e.g. shopping, cleaning).

### Feasibility

The group rounds could be carried out as planned, there were only very few technical difficulties with the online implementation. Most participants stated that no technical check was necessary prior to the sessions.

77% of all patients participated in at least 6 group sessions. 6 patients participated in 4 or less group sessions. The reasons given by participants were that they subjectively did not see any benefit for themselves by participating (n=3), 2 patients were unable to participate more often due to professional appointments, and one patient left for an inpatient rehabilitation for PCS. These participants did not differ significantly in terms of baseline characteristics, but were on average slightly younger (40.7 yrs ± 12.1, p=0.16) and stated a shorter duration of illness (9.7 month ± 7.1, p=0.20).

### Evaluation of the group content

Patients rated the various therapeutic modules as mostly helpful with some differences between the groups (Face-to-Face/Online). Results are shown in **Table 2**.

**Table 2:** Evaluation of the group content.

How much did you benefit from the following contents of the group therapy?**	total n=42	online n=25	Face-to-face n=17	p
Exchange with others who are suffering from PCS	9.0 (7.75-10.0)^a^	9.0 (7.5-10.0)	9.0 (7.5-10.0)	0.365
Relaxation techniques/techniques for stress regulation	6.0 (3.75-8.0)	6.0 (3.5-7.5)	7.0 (3.5-8.5)	0.408
Involvement of experts	9.0 (6.75-10.0)	8.0 (6.5-9.0)	9.0 (5.5-10.0)	0.345
Dealing with exhaustion/limited energy levels	7.0 (4.75-9.0)	6.0 (4.0-8.0)	9.0 (6.0-9.5)	0.048*
Networking with others who are suffering from PCS	8.0 (5.75-10.0)	8.0 (6.5-10.0)	8.0 (4.5-9.5)	0.449
Knowledge on the topic of PCS	8.0 (5.0-9.0)	7.0 (4.0-9.0)	8.0 (5.5-10.0)	0.265
Activation of personal resources	7.0 (3.75-8.25)	6.0 (2.0-8.0)	8.0 (6.0-9.5)	0.028*

**0-10 not helpful at all…. 10= Very helpful; ^a^ median (percentile-range 25-75) *p-values considered statistically significant.

83% of participants would recommend the group to friends (94% face-to-face; 77% online; p=0.04). When asked whether relatives of affected PCS patients should also receive such an offer (in a group or as individual counselling), 63.4% answered yes (58.8% face-to-face; 65.4% online; ns).

### Qualitative analysis of the feedback

At the end of the questionnaire, patients had the opportunity to add free text and provide feedback on the group (“How do you rate the group program? What would you like to report back?”).

The inductively emerged category ´appreciation of the group leaders´ was commented on most frequently (total 42%); participants emphasized ‘the creation and promotion of the pleasant atmosphere and the very appreciative attitude’ and the ´very empathetic moderation´ and ´being taken seriously` with the complaints (online group participants). The second most frequent statements (total 28% each) were made in the categories according to Yalom ´group cohesion´ and ´exchange of experiences and information´. Examples include ‘To get understanding from those who are similarly affected, you then have less of the need to always have to explain yourself to other people’, ‘I feel understood and part of a community´ or, that ´Exchange with other participants´ was particularly helpful.

**Figure 1:**
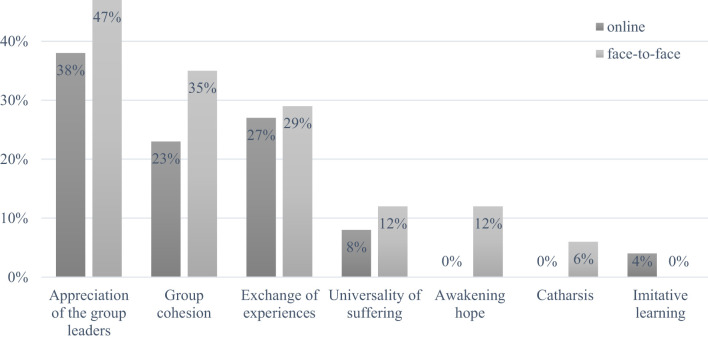
Frequency of comments according to Yalom’s effectiveness factors for group therapy.

The categories and the frequency of comments within each category are shown in **Figure 1**. Other comments in the free text fields (not shown) could be assigned to the categories ´universality of suffering´, ´awakening hope´, ´catharsis´ and ´imitative learning´.

### Subjective assessment of symptom severity/development

**Figure 2:**
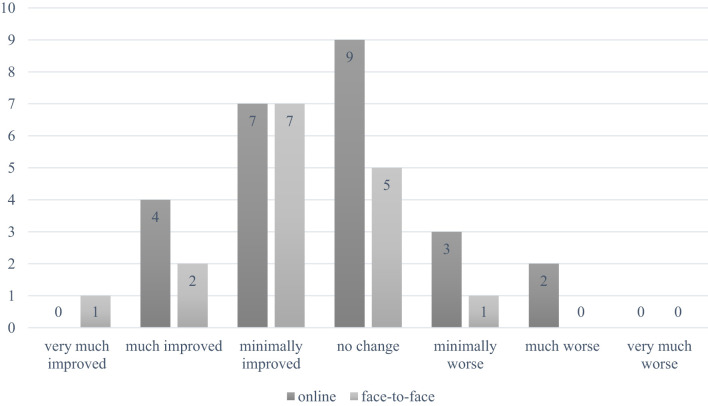
Patients global impression of change.

A global assessment of symptom change revealed that 62.6% of participants in the face-to-face group and 34% in the online group reported at least a slight improvement (**Figure 2**). Conversely, 6.3% of the face-to-face group (n=1) vs. 20% of the online group (n=5) indicated a deterioration in their condition compared to the initial assessment (ns).

All patients, including those who reported a worsening of their condition, were offered follow-up appointments at the clinic conducting the intervention. These sessions provided an opportunity for individualized assessment and counseling. Based on their needs, patients received tailored recommendations for further therapeutic interventions or referrals to specialized services.

## Discussion

Recent studies have indicated that up to 10% of patients continue to experience symptoms of PCS for weeks and months or even years following SARS-CoV-2-infection. The provision of group therapy as a supportive intervention for the often mentally burdened patients is a logical approach that could be made available to a greater number of those affected. The aim of the study presented here was to develop such a concept and to test it for acceptance and feasibility.

Acceptance and demand for the group were high, with the majority of patients (77%) participating in six or more group sessions. In their personal feedback, the majority of patients reported that participation was strenuous and occasionally led to significant exhaustion. However, the experience of the group intervention was perceived as beneficial, leading to continued participation. Additionally, no other adverse effects of group participation were reported.

When assessing the global impression of change, only six out of 43 participants reported a worsening of their condition, five of whom participated in the online group. This indicates that the concept is, on the whole, a safe and reliable therapy option. As there is considerable uncertainty among patients, patient groups, and even clinicians regarding the potential harms and overwhelming nature of psychotherapy or psychoeducational programs for PCS patients, this is an important finding.

In our group of patients, the proportion of patients who reported deterioration is within the anticipated range of adverse effects in group therapy (29). Similar results regarding feasibility and safety were also shown by Schilling et al., McGregor et al. and Kuut et al., who successfully conducted psychoeducational-oriented and cognitive-behavioral online groups and online mental health groups for patients suffering from PCS (29).

A comparison of the two modes of implementation with respect to the global impression of change, it can be seen that participants in the face-to-face group reported improvement more frequently than those in the online group. Specifically, 62.6% of participants in the face-to-face group and 34% in the online group reported feeling at least slightly better. However, due to the significantly longer duration of the illness, the patients in the online group had different initial conditions, and it can be assumed that there is progressive chronification, which is known to lead to an ever-decreasing response to psychotherapeutic interventions (30–32).

In addition to these *a priori* differences between the groups, these results are also remarkable because one might expect that the risk of being overwhelmed would be greater in face-to-face formats than in online formats. However, this was not the case, indicating that the effort required to participate in the sessions did not appear to worsen symptoms. In fact, none of the patients stated in their personal feedback that a worsening of their symptoms in their subjective perception was due to group participation.

Besides this the group content was predominantly rated positively. Patients reported that exactly those topics were discussed that play an important role in the context of PCS and the associated health, social and psychological consequences. The two group modes differed only in the evaluation of the two topics ´dealing with exhaustion´ and `activation of resources´. A longer duration of illness, less mobility and disease-related restrictions in the online-group may be a reason for this. It is likely that communicating about topics that touch on very personal aspects of coping with illness reaches its limits in the online setting (33). It cannot be ruled out that there were other differences between the groups that were not recognized or recorded (by PCS score), but which played a role in the result and the response to the group intervention.

A look at the qualitative evaluation of the patient comments shows that the attitude of the leaders and the guidance was perceived as particularly important (“found it helpful that conversations were accompanied by doctors and psychologists”). Surprisingly, it was not the well-known factors of group psychotherapy that were frequently highlighted (sense of belonging, positive feedback), but the appreciation of the group leaders was mentioned most frequently. This may suggest that individuals with PCS, who frequently feel dismissed by medical professionals due to the persistent and distressing symptoms and the lengthy process of diagnoses, find the guided group particularly beneficial. The group thus facilitates a corrective relationship experience in the medical system and thus the freedom to engage in further supportive therapies.

## Limitations and strength

The results presented here provide information on the feasibility and acceptance of the therapy concept. Due to the lack of control conditions, no statement can be made regarding efficacy. The patients were also not randomly assigned to a form of therapy. It is now necessary to test the potential effectiveness of the therapeutic concept under control conditions.

In contrast, it is a strength of the study that it was conducted under naturalistic conditions, so that a statement can be made on the feasibility, acceptance and practicability under realistic clinical circumstances. It is a further strength that it could be shown that supportive therapy can also be made available to patients who are unable to visit a clinic or practice due to their limitations. It is also a strength that patients themselves were able to have their say and we were able to incorporate their statements on this new form of therapy into the analysis.

## Conclusion for practice

In conclusion, an outpatient group therapy for individuals with PCS can be conducted in both online and a face-to-face formats. There was a considerable demand for online group therapy. Patients with severe symptoms and limited mobility are looking for remote therapeutic services that can be accessed from home, which is often the only feasible option for accessing such supportive therapy. Even though differences between face-to-face and online formats must be taken into account, both formats are perceived as feasible, save and highly valued by patients who are willing to get psychosocial/supportive therapy, who would like to contribute their own topics and to benefit from each other´s experiences. It is essential to clarify the openness to such an exchange during the screening assessment.

## Data Availability

The data set analyzed for this study is available on request from the corresponding author.
