# Supplementary material for: Outpatient group therapy for post-COVID patients - a naturalistic feasibility study of a face-to-face and online group concept
Source: Front Psychiatry. 2024 Dec 17;15:1500210. doi: 10.3389/fpsyt.2024.1500210 (PMC11687220; doi:10.3389/fpsyt.2024.1500210)
Supplement: Supplementary file 1 [file Table1.docx]

**Outpatient group therapy for post-COVID patients - a naturalistic feasibility study of a face-to-face and online group concept**

*Supplement*: Treatment concept overview

| Session | Thematic focus | Session content |
| --- | --- | --- |
| 1 | Welcome session | - Welcome and introduction of the group leaders. - Explanations on the duty of confidentiality, explanations on the procedure and the general group rules, mutual introductions. - Outlook on the planned topics. - All patients have the opportunity to introduce themselves and share aspects of their experience with PCS with other patients. - “3-minute-Breathing-Space” exercise |
| 2 | Stress, illness-related strains | - Interaction of stress and symptoms; possible influence of physical, mental or emotional stress factors on various symptoms. - Presentation of various techniques for stress regulation (e.g. Progressive Muscle Relaxation, Mindfulness Based Stress Reduction – “Body Scan”, different imagination exercises – “Safe Place” or “Shedding burdens”). - Space for open exchange between participants. - Imagination exercise (“Safe Place”) |
| 3 | Knowledge about PCS | - Information on PCS and the current status of treatment options. - Patients have the opportunity to ask their own questions to a physician specialized in PCS. - *Note: the content of this session needs to be continuously adapted to the most recent scientific evidence.* - Space for open exchange between participants. - “3-minute-Breathing-Space” |
| 4 | Energy management | - Interim conclusion and debriefing of the third session, what other questions have arisen? - Psychoeducational input on dealing with limited energy reserves (“Pacing”) and stress intolerance; ideas for an individual energy management; encouragement to write down helpful ideas/successful strategies every day (“helpful strategies diary”). - Space for open exchange between participants about their individual strategies on energy management. - “3-minute-Breathing-Space” |
| 5 | Resources, coping strategies | - Existing resources (despite illness) and possibilities for using existing resources and/or activating them. - Collection of ideas: in which areas of life are there resources that may be utilised during this phase of the illness? What helps me or what prevents me from drawing on these resources? - Space for open exchange between participants about their individual ideas on existing resources and their activation. - “3-minute-Breathing-Space” |
| 6 | Changes in the various areas of life | - Psychoeducation on social roles, expectations and needs with a focus on role changes in the workplace, role expectations in the family and potential conflicts arising from this. - What possible solutions/communication channels can be developed? - Space for exchange on participants experiences and their ways of dealing with these changes. - “3-minute-Breathing-Space” |
| 7 | Physical resilience and breathing techniques | - Session together with physiotherapist - instructions on breathing exercises and exercises to increase physical resilience (duration approx. 25 min, participation depending on personal fitness). - Opportunity to discuss questions with the physiotherapist about patients possibilities for increasing physical training. - Space for exchange between participants: what possibilities for - even minor - physical exertion have the participants already been able to develop? What could be possible again in the future? - “3-minute-Breathing-Space” |
| 8 | Closing session | - Conclusion and outlook. - Space for exchange between the participants about their experiences of the last few weeks - have there been any changes, if so, what has changed? - Feedback of and to the therapists. - “3-minute-Breathing-Space” |
| 9 | Refresher session  (after 8 weeks) | - Review of the course of symptoms over the last few weeks; What was helpful recently? What did not help? - Space for exchange between the group participants. - At the patient's request, further psychoeducational input on the topics already mentioned. - “3-minute-Breathing-Space” |
